# Rare Case of Male Pelvic Actinomycosis

**DOI:** 10.5334/jbsr.1387

**Published:** 2018-01-31

**Authors:** Johan Dehem, Stijn Deloose, Christian Feys

**Affiliations:** 1 Jan Yperman Ziekenhuis, BE

**Keywords:** Actinomycosis, ADC, pseudotumor, microabcesses, infection, pelvic mass

A 51-year-old male with three weeks of increasing back pain, sciatalgia, and recent fever spike searched medical attention. Clinical examination highlights positive Lasègue and tender right fossa. Laboratory testing returns high C reactive protein and leucocytosis.

**Figure 1:**
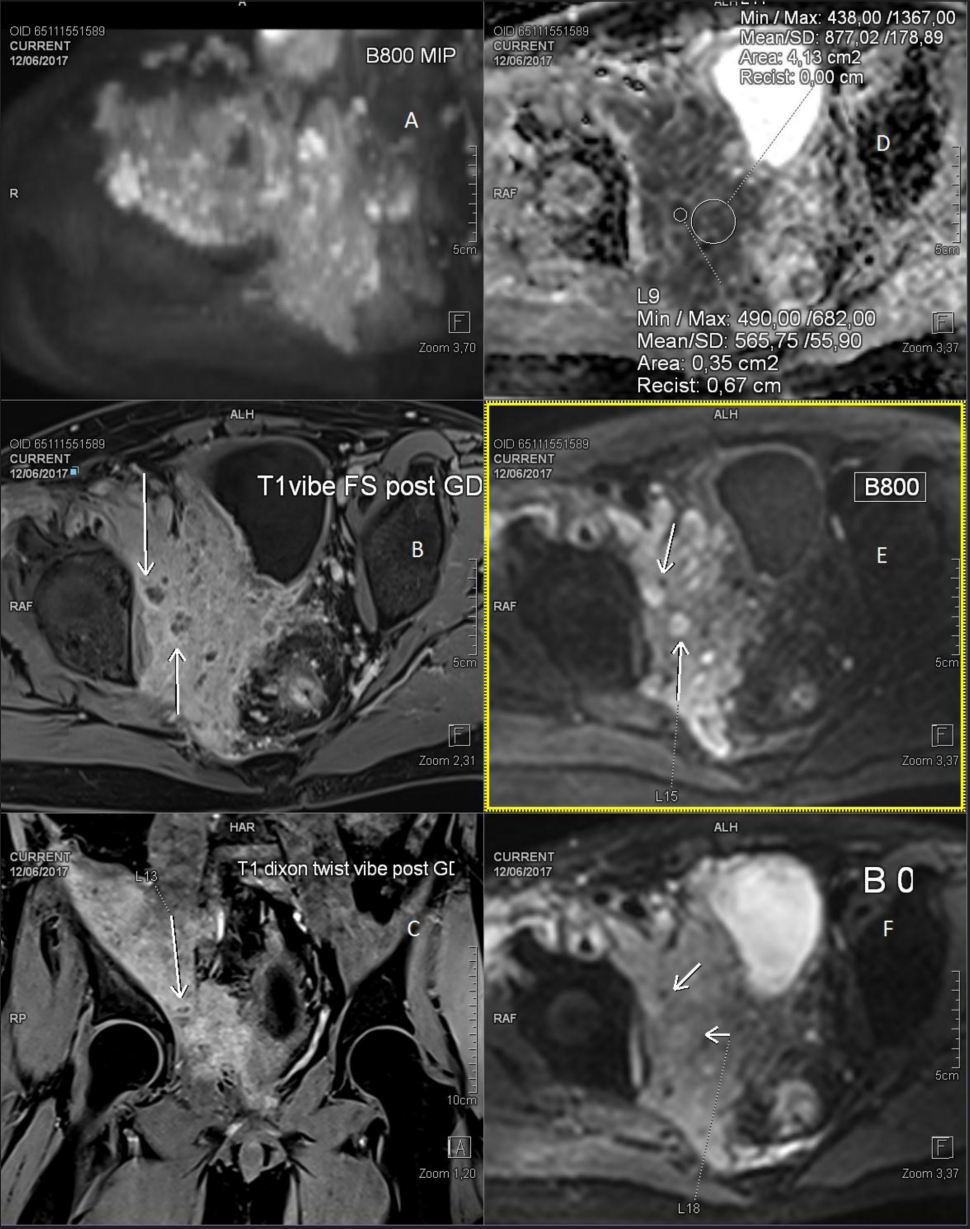
MRI at initial presentation with T1 fatsat images post gado **(B, C)** and DWI images **(A, D, E, F)**.

Pelvic MRI reveals an ill-defined large enhancing overall low ADC mass (Figure 1D, larger ROI) with smaller non-enhancing pockets (arrows in Figure 1B, C) with very low ADC:566 (Figure 1D, small ROI) and high signal on b-800 images (Figure 1E, arrows). They are hard to see on low b-value images (Figure 1F, arrows), but they stand out on Maximum Intensity Projection b-800 image (Figure 1A). Note the aggressive crossing of fat planes and invasion of bladder and piriform, iliac and obturator muscle. There are no ascites nor enlarged lymph nodes. Differential diagnosis included malignancy versus pseudotumor or mass-like infection. Chest CT (not shown) demonstrates nodules in both lungs and left lower lobe patchy air-space consolidation, which might suggest fungal or atypical bacterial infection, e.g. Nocardia or Actinomycosis rather than metastatic disease.

**Figure 2:**
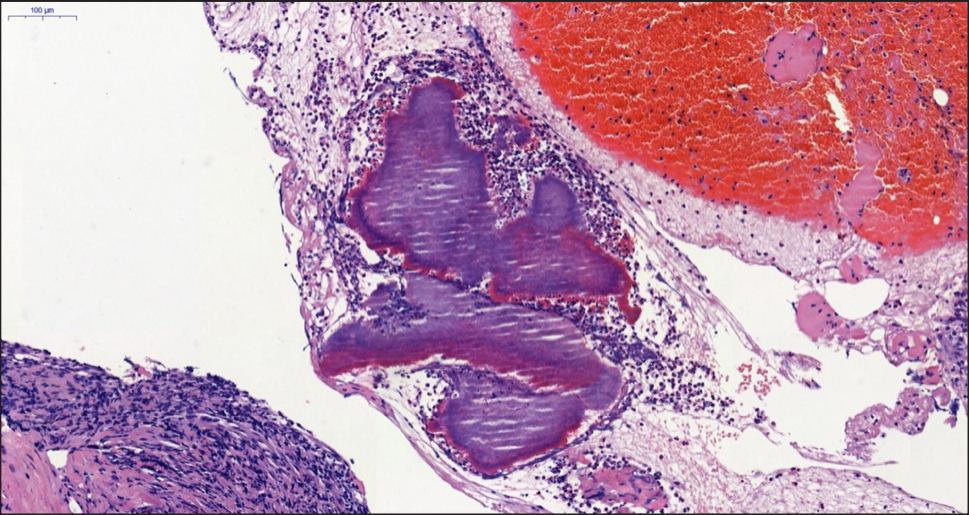
Formalin fixed and paraffin embedded HE-stained section.

Frozen sections on surgical tissue samples (anterior to the right anterior iliac crest) reveals connective tissue, fat, muscle, and inflamed tissue without malignancy. Formalin fixed and paraffin embedded HE-stained sections (Figure 2) shows striking deposition of star-like eosinophilic material round blue filamentous bacteria (Splendore-Hoeppli phenomenon), which is pathognomonic for Actinomyces.

**Figure 3:**
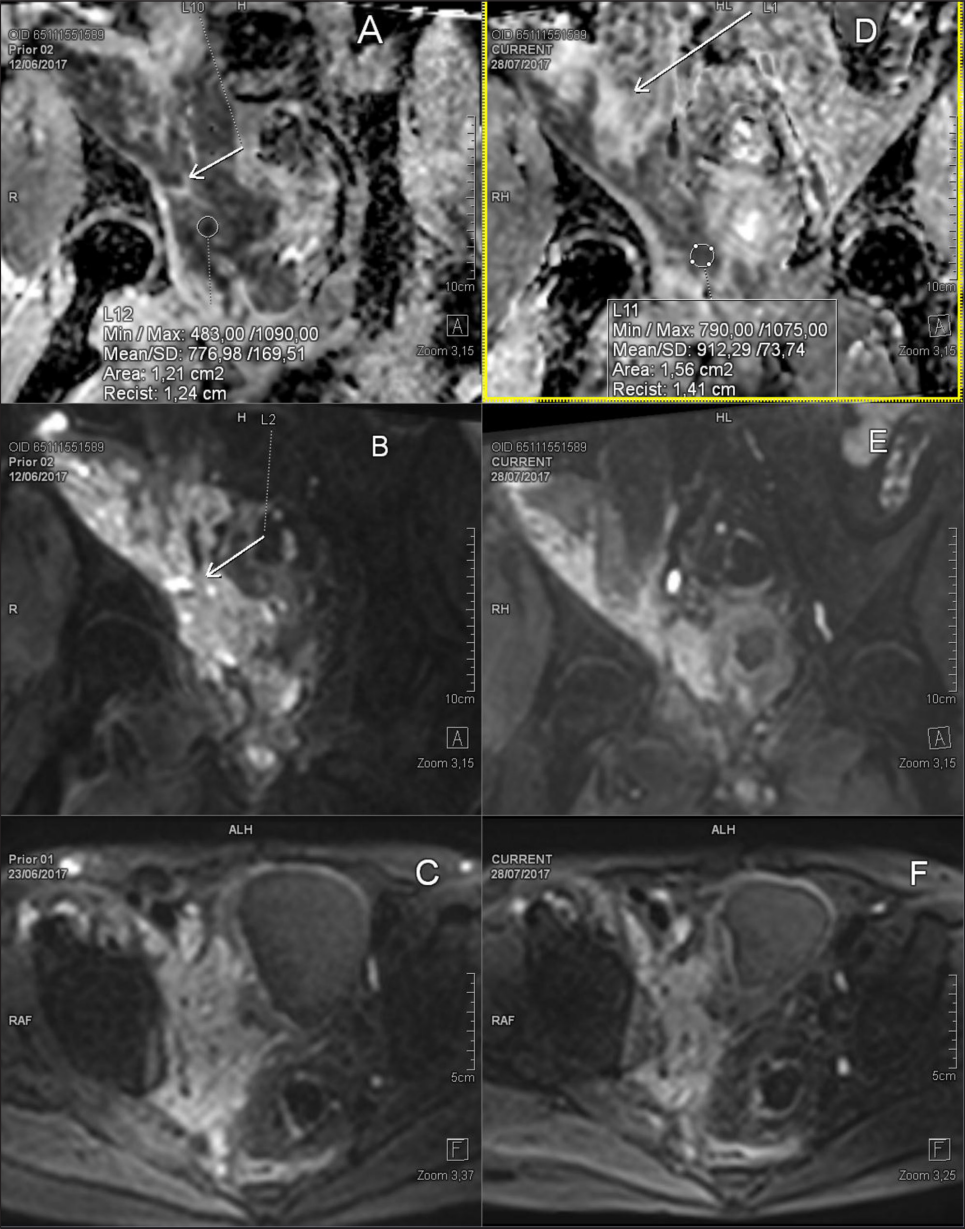
DWI images at initial presentation **(A, B, C)** and after six weeks of antibiotics **(D, E, F)**.

The match of radiology and pathology findings is consistent with pseudotumoral Actinomycosis. Treatment with Amoxicillin/clavulanic acid results in pain relief and clear clinical and biochemical improvement. After six weeks of treatment there is marked volume loss of the mass and better delineation of bladder and psoas muscle (Figure 3; pre- vs post-treatment MRI). ADC values in the residual mass are higher after treatment (ROI measurement Figure 3D versus Figure 3A). The pus containing pocket (Figure 3A, B arrow) has disappeared (Figure 3E) after treatment.

Actinomycosis is a rare, chronic, destructive, invasive gram-positive bacterial infection. It has been reported as odontogenic, in the head and neck (mandibular osteomyelitis) and thoracic region. Abdominal, pelvic actinomycosis is even more rare and mainly found in immunocompromised patients. In women, IUCD-associated pelvic actinomycosis has been reported. Our patient, however, had no medical history except for orchitis treated three months ago. Without clinical tell-tale signs, e.g. cutaneous sinus tracts, diagnosing actinomycosis can be a challenge. Aggressive pseudo-tumor presentation and hematogenous spread to the central nervous system and lungs is possible [1].

Suggesting the diagnosis of actinomycosis early is vital in patient management: timely antibiotics do cure the patient. Besides clinical and laboratory findings of inflammation, imaging features can provide us with a few hints. Actinomycosis is highly aggressive, infiltrating tissues, crossing fatplanes, and can invade muscles, organs, bones, and joints. Small non-enhancing microabcesses with striking low ADC might be a clue to look for. During treatment, measuring ADC values allows for non-invasive monitoring of pus content in the micro-abcesses.
